# Wellness profile of residents in urban retirement villages in the City of Johannesburg

**DOI:** 10.4102/hsag.v30i0.3002

**Published:** 2025-09-02

**Authors:** Lynn Smith, Simone Ferreira, Thembisile Mbatha

**Affiliations:** 1 Department of Sport and Movement Studies, Faculty of Health Sciences, University of Johannesburg, Johannesburg, South Africa

**Keywords:** biokinetics, community health, elderly health status, good health and wellbeing, healthcare, physical wellbeing, retiree health trends

## Abstract

**Background:**

Retirement is a significant life transition that often brings changes in daily routines, including health screening and levels of physical activity.

**Aim:**

The aim of this study was to determine the wellness profile of residents living in retirement villages.

**Setting:**

This study was conducted in the City of Johannesburg.

**Methods:**

This cross-sectional, descriptive and quantitative study included 108 retirees from 7 retirement villages. To determine the wellness profile, biokinetic wellness screening tests were performed, including blood pressure, heart rate, rate pressure product, height, weight, body mass index (BMI), handgrip strength, sit-and-reach test and Apley’s back scratch test.

**Results:**

The median age of the sample was 75 years. Significant variability was noted in the BMI measurement (25.35 kg/m^2^; IQR: 6.83), and the median rate pressure product (RPP) was 10 284 mmHg/min (IQR: 2962). Data revealed limited flexibility for Apley’s scratch test (-34; -31) and sit-and-reach test (0.00).

**Conclusion:**

Strength was greater among male participants, while better flexibility but higher heart rates were recorded among females. Tailored interventions emphasising cardiovascular health, weight management and physical performance are recommended among retirees.

**Contribution:**

This resource will guide policymakers, community managers and healthcare professionals in designing targeted interventions and inform future research on ageing and wellness in urban settings.

## Introduction

Research indicates that despite availability of health screenings in a global context, participation by the elderly population in these screenings has not increased (Xu, Pan & Li 2024). In South Africa, evidence exists that lower pensions are associated with poorer health outcomes, affecting those from low- to-middle-income countries (LMICs) and less affluent communities (Lloyd-Sherlock & Agrawal 2014). The City of Johannesburg (CoJ) in South Africa encompasses low-income residential areas that have undergone various changes over the years (Abrahams & Everatt 2019). This region is shaped by diverse residents, who come from a range of cultural and socioeconomic backgrounds, faced with challenges such as economic disparities, limited resources and infrastructural needs (Johnson & Patel 2016). Older adults living in retirement villages within the CoJ are particularly vulnerable to these challenges, as ageing is often accompanied by health concerns that require adequate support systems. Although local initiatives and community projects are actively working to address these issues and improve the overall quality of life for its residents (Nguyen & Ross 2021), access to health screenings and services remains a challenge.

Assessing the wellness profiles among retired individuals in the CoJ is essential for understanding their health and wellbeing, and identifying opportunities for intervention and enhancement of healthy lifestyles. Several factors may influence wellbeing in this region, such as access to recreational facilities, safety concerns, socioeconomic status and community support (Brown 2022; World Health Organization [WHO] 2023). Cardiovascular health is a significant concern among older adults in South Africa, with hypertension being highly prevalent in this population. Gender-specific trends in cardiovascular health indicate that while men typically have higher systolic blood pressure (SBP), women tend to display higher diastolic blood pressure (DBP) and resting heart rates, which are critical indicators of cardiovascular strain (Whelton et al. 2022). Alongside cardiovascular concerns, there has been a notable shift in the prevalence of overweight and obesity among the elderly. It was found that the proportion of elderly women who were overweight or obese increased significantly (Egal 2015; Owolabi et al. 2017), while the prevalence among elderly men decreased substantially from 66.7% to 42.8% (Egal 2015).

In the South African context, access to healthcare in safe neighbourhoods significantly influences health awareness. Crime and perceived neighbourhood safety have been linked to fear of walking, specifically as a form of physical activity (He et al. 2020). In addition, retirees from lower-income backgrounds often face greater challenges in accessing healthcare and maintaining a healthy lifestyle because of financial constraints. Limited resources make it difficult for older adults to afford medical check-ups, nutritious food and safe spaces for exercise, increasing their risk of chronic conditions such as hypertension and diabetes (Goetjes et al. 2020; Yang et al. 2023). However, group physical activities, such as walking clubs can help retirees build social connections, combat isolation and create a sense of belonging (Gonnord et al. 2023).

Collaborative efforts involving researchers, healthcare providers, community leaders and local organisations are crucial for designing effective strategies to promote physical activity and enhance the wellbeing of retirees in the CoJ. Potential interventions that would contribute to Sustainable Development Goal 3 (Good Health and Wellbeing) could include the development of safe and accessible health clinics and recreational spaces for community-based wellness, exercise programmes and education tailored for older adults’ health (Lee, Park & Kim 2024).

The aim of this study was to conduct biokinetic wellness screening with the purpose of establishing a wellness profile among retired individuals residing in the CoJ retirement villages.

## Research methods and design

### Study design

The research design was cross-sectional. Descriptive and quantitative data were collected.

### Site of study and sampling

Data were collected at seven retirement villages, located in the CoJ, South Africa. The research participants were residents in retirement villages in the CoJ who were recruited through purposive sampling. Out of a population of 142 retirees, the sample size for this study was 108.

### Selection and recruitment of participants

Prior to testing of the participants, contact was made with the region’s area managers, who granted gatekeeper permission. The area managers communicated to the residents at the various retirement villages that research would be conducted. The researchers furnished residents with an information letter that explained the purpose of the research, their rights as participants as well as the risks and benefits associated with participation. Before testing took place, the researchers obtained consent from each participant whose data were utilised for this study.

### Measuring tools and data collection

The researchers performed seven biokinetic wellness tests, namely resting blood pressure, resting heart rate, weight, height, sit-and-reach test (Wells & Dillon 1952), Apley’s back scratch test (Heinecke, Thuesen & Stow 2014) and handgrip strength (HGS). Rate pressure product was calculated from the resting blood pressure and resting heart rate obtained, while body mass index (BMI) was calculated for the weight and height measurements. For consistency, reliability and validity, each researcher was responsible for conducting certain tests and conducted those same tests on all participants. Each participant’s results were recorded on a data collection sheet which was used for comparison against established age-appropriate normative values for the purpose of this research. After testing, the researchers provided education and feedback to the participants regarding their biokinetic wellness screening results, and each participant received a generic exercise programme.

### Data analysis

The data collected were quantitative and were captured and coded in a Microsoft Excel spreadsheet. Participant names or identifying information were replaced and only codes were assigned. The data were analysed on the Statistical Package for Social Sciences (SPSS) version 29 (IBM, Armonk, New York, United States). The Kolmogorov-Smirnov and Shapiro-Wilk tests were computed to determine if the data were normally distributed. Because of the data not being normally distributed, the median and interquartile ranges (IQRs) are reported.

### Ethical considerations

Permission to conduct this research was obtained from the Faculty of Health Sciences Research Ethics Committee of the University of Johannesburg (reference no: REC-3116-2025). All procedures performed during this study were in accordance with the ethical standards of the institutional research committee and with the 1964 Helsinki Declaration and its later amendments or comparable ethical standards. Written consent was obtained from all participants, and confidentiality was maintained throughout.

## Results

The descriptive statistics of the female participants presented in Table 1, reveal a predominantly older population, with a median age of 75 years (IQR: 10.25 years), ranging from 61 to 92 years. Median SBP (135 mmHg) and DBP (79 mmHg) fall within the prehypertensive range, with some participants showing elevated values, suggesting potential cardiovascular risks. The median heart rate of 80 bpm (IQR: 16.50) is within normal resting limits, although the upper range (102 bpm) indicates elevated levels in certain individuals. The median RPP of 10 720 mmHg/min (IQR: 3606) reflects moderate myocardial workload, with variability across the group.

**TABLE 1:** Descriptive statistics of the female participants.

Variable	Median	IQR	Minimum	Maximum
Age	75.00	10.25	92.00	61.00
SBP (mmHg)	135.00	28.00	202.00	104.00
DBP (mmHg)	79.00	14.00	110.00	50.00
HR (bpm)	80.00	16.50	102.00	64.00
RPP (mmHg/min)	10720.00	3606.00	13120.00	6656.00
Weight (kg)	61.75	18.75	89.00	32.50
Height (m)	1.54	0.1175	1.75	1.38
BMI (kg/m^2^)	25.75	6.05	37.40	12.70
Handgrip strength_L (kg)	15.45	7.125	24.10	9.30
Handgrip strength_R (kg)	17.25	9.30	27.30	8.00
Apleys scratch_L (cm)	−28.00	−18.25	0.00	−68.00
Apleys scratch_R (cm)	−28.00	−16.75	0.00	−62.00
Sit-and-reach_L (cm)	0.00	−13.25	0.00	−48.00
Sit-and-reach_R (cm)	0.00	−10.375	1.00	−42.00

IQR, Interquartile Range; SBP, Systolic Blood Pressure; DBP, Diastolic Blood Pressure; HR, Heart Rate; RPP, Rate Pressure Product; mmHg, millimetres of mercury; bpm, beats per minute; mmHg/min, millimetre of mercury per minute; kg, kilogram; m, meter; BMI, Body Mass Index; kg/m^2^, kilogram per meter squared; cm, centimetre.

Anthropometric and physical fitness measures highlight key characteristics and challenges. The median BMI of 25.75 kg/m^2^ indicates an overweight population, with values spanning from underweight (12.70 kg/m^2^) to obese (37.40 kg/m^2^). Handgrip strength was higher on the right hand (median: 17.25 kg) than the left (15.45 kg), but both measures suggest moderate muscle strength. Flexibility tests, including Apley’s scratch and sit-and-reach, show median values at or below zero, indicating limited flexibility, particularly in the lower back, hamstrings and shoulders.

Descriptive statistics for the male participants are summarised in Table 2. The median age was 75 years (IQR: 7.25 years), with participants ranging from 52 to 95 years old. Systolic blood pressure had a median value of 137 mmHg (IQR: 25 mmHg), while DBP had a median of 74 mmHg (IQR: 24 mmHg). Resting heart rate showed a median of 76.5 bpm (IQR: 18 bpm), and the RPP, an indicator of myocardial workload, had a median value of 10 704 mmHg/min (IQR: 2902 mmHg/min). Regarding anthropometric measures, the median weight and height were 75.05 kg and 1.655 m, respectively, resulting in a median BMI of 26.1 kg/m^2^ (IQR: 7.8 kg/m^2^), indicative of a range from normal weight to obesity.

**TABLE 2:** Descriptive statistics of the male participants.

Variable	Median	IQR	Minimum	Maximum
Age	75.00	7.25	95.00	52.00
SBP (mmHg)	137.00	25.00	182.00	98.00
DBP (mmHg)	74.00	24.00	110.00	50.00
HR (bpm)	76.50	18.00	102.00	60.00
RPP (mmHg/min)	10704.00	2902.00	14852.00	6832.00
Weight (kg)	75.05	29.65	128.90	49.00
Height (m)	1.655	0.1225	1.86	1.55
BMI (kg/m^2^)	26.10	7.80	42.60	18.00
Handgrip strength_L (kg)	24.20	8.40	45.40	21.40
Handgrip strength_R (kg)	26.50	5.80	47.00	25.40
Apleys scratch_L (cm)	−36.00	20.00	0.00	−67.00
Apleys scratch_R (cm)	−31.00	21.625	0.00	−92.00
Sit-and-reach_L (cm)	−4.50	14.75	3.00	−32.00
Sit-and-reach_R (cm)	−5.20	16.625	0.00	−32.00

SBP, Systolic Blood Pressure; DBP, Diastolic Blood Pressure; RPP, Rate Pressure Product; IQR, interquartile range; BMI, body mass index; HR, Heart Rate; mmHg, millimetres of mercury; bpm, beats per minute; mmHg/min, millimetre of mercury per minute; kg/m^2^, kilogram per meter squared; m, meter; kg, kilogram; cm, centimetre.

Physical performance metrics revealed variations in flexibility and strength among participants. Median HGS was 24.2 kg (IQR: 8.4 kg) for the left hand and 26.5 kg (IQR: 5.8 kg) for the right hand, suggesting stronger dominant hand performance. For flexibility, median Apley’s scratch test scores were –36 cm (IQR: 20 cm) for the left side and –31 cm (IQR: 21.625 cm) for the right. Sit-and-reach test results showed a median of –4.5 cm (IQR: 14.75 cm) for left side and –5.2 cm (IQR: 16.625 cm) for the right side, indicating limited forward flexibility. These results highlight notable variability in both cardiovascular and musculoskeletal measures across the cohort.

### Comparison between genders

The Kolmogorov-Smirnov test was conducted to assess normality, and as the data did not follow a normal distribution, the Mann–Whitney *U* test was used for inferential statistics (see Table 3).

**TABLE 3:** Inferential statistics of variables between genders.

Variable	*p*	Significance (*p* < 0.05)
Age	0.718	Not significant
SBP (mmHg)	0.403	Not significant
DBP (mmHg)	0.490	Not significant
HR (bpm)	0.922	Not significant
RPP (mmHg/min)	0.528	Not significant
Weight (kg)	0.004†	Significant
Height (m)	< 0.001†	Highly significant
BMI (kg/m^2^)	0.657	Not significant
Handgrip strength_L (kg)	< 0.001†	Highly significant
Handgrip strength_R (kg)	< 0.001†	Highly significant
Apleys scratch_L (cm)	0.063	Not significant
Apleys scratch_R (cm)	0.043†	Significant
Sit-and-reach_L (cm)	0.215	Not significant
Sit-and-reach_R (cm)	0.055	Not significant

RPP, Rate Pressure Product; SBP, Systolic Blood Pressure; DBP, Diastolic Blood Pressure; HR, Heart Rate; BMI, body mass index; mmHg, millimetres of mercury; bpm, beats per minute; mmHg/min, millimetre of mercury per minute; m, meter; kg/m^2^, kilogram per meter squared; kg, kilogram; cm, centimetre.

† , The significance level is 0.050.

No significant difference in *age* was observed between male and female participants (*p* = 0.718). Both groups had a median age of 75 years, which is consistent with an older adult population (60–95 years) commonly seen in health studies. No significant difference was found in *SBP* between males and females (*p* = 0.403). Median SBP values were 135 mmHg for females and 137 mmHg for males, which aligns with the prehypertension range common in older adults. *Diastolic Blood Pressure* did not differ significantly between genders (*p* = 0.490). Median DBP values were 79 mmHg for females and 74 mmHg for males, both near the prehypertension range for older adults. No significant gender difference was observed for *HR* (*p* = 0.922), with median values of 80 bpm for females and 76.5 bpm for males, both within the normal resting heart rate range. No significant difference in *myocardial workload* (RPP) was found between males and females (*p* = 0.528). Median RPP values were 10 720 mmHg/min for males and 10 704 mmHg/min for females, indicating moderate myocardial strain typical of older adults. A significant difference in *weight* was found (*p* = 0.004), with median values of 75.05 kg for males and 61.75 kg for females. This aligns with normative data showing that males generally weigh more than females. *Height* proved a significant difference (*p* < 0.001), with median values of 1.65 m for males and 1.54 m for females, consistent with expected gender differences in body size. No significant difference in *BMI* was noted between males and females (*p* = 0.657). Median BMI values for both groups fell within the overweight range (25.75 kg/m^2^ for females and 26.1 kg/m^2^ for males), typical in older populations.

Significant gender differences in *HGS (Left and Right)* were observed for both hands (*p* < 0.001 for both). Median values for left-hand grip strength were 24.2 kg for males and 15.45 kg for females, while right-hand values were 26.5 kg for males and 17.25 kg for females. This aligns with established norms showing males generally have greater muscle strength. No significant gender differences were found in the *Apley’s Scratch Test (Left and Right)* for left shoulder flexibility (*p* = 0.63), although a significant difference was observed in right shoulder flexibility (*p* = 0.043), suggesting slightly better flexibility in males on that side. Both groups showed limited flexibility, consistent with normative data for older adults. *Sit-and-Reach Test (Left and Right)* proved no significant differences for either the left side (*p* = 0.215) or right side (*p* = 0.055), although the latter approached significance. Both groups demonstrated limited flexibility, common in older populations.

Significant gender differences were found in weight, height, HGS (both hands), and right Apley’s scratch flexibility. No significant differences were observed for age, SBP, DBP, HR, RPP, BMI, left Apley’s scratch flexibility, or sit-and-reach tests. These findings align with established norms for older adults, particularly regarding gender differences in body composition and muscle strength.

## Discussion

The male and female participants’ data reveal key insights into the health and physical capabilities of older adults, highlighting similarities and differences in ageing patterns between genders. Both groups had a median age of 75 years, reflecting the ageing population in South Africa. However, the broader IQR for women (10.25 years) compared to men (7.25 years) suggests greater variability in the age distribution among women, potentially because of their longer life expectancy (WHO 2021b). These findings align with global trends, where women typically outlive men, experiencing both benefits and challenges associated with longevity (Gerontological Society of America 2022; WHO 2021a). In the South African context, there is limited research providing comprehensive details on the gender gap in life expectancy between genders; however, international research has cited risky behaviours of males and the more frequent utilisation of health services among females as common reasons (Fleming & Agnew-Brune 2015; South African Medical Research Council 2021).

In terms of cardiovascular health, both groups exhibited elevated SBP and DBP, consistent with the high prevalence of hypertension in older populations (Whelton et al. 2022). Men had slightly higher median SBP (137 mmHg) compared to women (135 mmHg), but women displayed a higher median DBP (79 mmHg vs. 74 mmHg). It is known that females tend to present with a lower SBP owing to increased oestrogen levels (Masi et al. 2009). Resting HR was higher among women (80 bpm) than men (76.5 bpm), which aligns with known gender differences in cardiovascular physiology. The rate pressure product, a measure of myocardial oxygen demand, was comparable between men (10 704 mmHg/min) and women (10 720 mmHg/min), suggesting similar cardiovascular workload. This is consistent with research demonstrating that elderly women have smaller left ventricular chambers resulting in higher resting heart rates and smaller stroke volumes (Yusifov, Woulfe & Bruns 2022). These findings emphasise the importance of monitoring cardiovascular health in both genders, with tailored strategies to address gender-specific risks.

Anthropometric measures revealed distinct differences between men and women. Men had higher median weight (75.05 kg) and height (1.655 m) compared to women (61.75 kg and 1.54 m, respectively). Despite these differences, the median BMI was similar for both groups, with men at 26.1 kg/m^2^ and women at 25.75 kg/m^2^, placing both genders in the overweight category. The wider range of BMI values among men (18.0 kg/m^2^ to 42.6 kg/m^2^) compared to women (12.7 kg/m^2^ to 37.4 kg/m^2^) highlights greater variability in body composition among men; although, it is important to note that BMI does accurately reflect body composition and fat distribution (ed. Callahan 2023). Overweight and obesity in both groups pose significant risks for chronic diseases such as diabetes and cardiovascular conditions (Sathian et al. 2024).

Physical performance metrics underscore notable gender differences in musculoskeletal strength and flexibility. Median HGS, a marker of muscle strength and function, was higher in men (24.2 kg left, 26.5 kg right) than in women (15.45 kg left, 17.25 kg right). This disparity aligns with established patterns of greater muscle mass and strength in men, although both groups displayed lower HGS than age-specific normative values (Dodds et al. 2014; Tomkinson et al. 2024). Flexibility, assessed via Apley’s scratch and sit-and-reach tests, showed limited ranges of motion in both genders, with negative median scores. Women demonstrated slightly better flexibility than men, particularly in the sit-and-reach test, which aligns with gender differences in joint range of motion (Stathokostas et al. 2013).

These findings emphasise the multifaceted nature of ageing, with both genders facing unique challenges. Men exhibited greater strength but higher weight variability, while women demonstrated slightly better flexibility but higher heart rates. Both groups would benefit from tailored interventions focusing on cardiovascular health, weight management and physical performance. Future research should explore longitudinal changes and the impact of targeted health programmes to promote functional independence and quality of life in older men and women.

### Recommendations

It is recommended that future research differentiate between various age brackets and genders to allow for more tailored interventions. In addition, structured physical activity programmes should be implemented for older adults, focusing on improving cardiovascular health, flexibility and strength through low-impact aerobic exercises, resistance training and stretching routines to enhance SBP, resting heart rate, HGS and overall flexibility. Regular health assessments should also be established to monitor key indicators such as blood pressure, resting heart rate and BMI, enabling early detection and management of cardiovascular strain and obesity-related risks while guiding individualised health plans to reduce potential complications.

## Conclusion

This study provides insight into the wellness profiles of retirees in the CoJ, South Africa. Similarities were noted in cardiovascular health, where elevated SBP and DBP and increased heart rates were prevalent across the sample. However, differences in weight, height and muscle strength were evident between male and female participants, highlighting the need for tailored health interventions. Both groups exhibited similar BMI values, placing them in the overweight category, underscoring the importance of weight management and chronic disease prevention in this population. Although males exhibited higher hand-grip strength, female participants demonstrated better flexibility but higher heart rates. This is aligned to established biological differences.

This profile serves as a valuable resource for policymakers, retirement community managers and healthcare professionals, enabling them to design and implement targeted interventions aimed at enhancing the overall quality of life and wellbeing of retirees. In addition, the study informs future research on ageing populations and the effectiveness of wellness initiatives in urban retirement settings.
